# The Lords' Committee

**Published:** 1891-05-09

**Authors:** 


### THE LORDS' COMMITTEE.

On Monday, April 27th, Mr. David Cannon, Secretary to the Hospital for Women, stated that it was the first of such special hospitals, and was founded in 1842 in Red Lion Square, and removed to Soho Square in 1851. It was incorporated by Royal Charter in 1887. All the premises were freehold, and, although there was a heavy mortgage, it was hoped by means of legacies to clear that off next year. The mortgage had been reduced from £18,300 to £10,800. There were sixty-six beds, of which fifteen were for patients who paid from 25s. to £2 2s. a week. Some of the in-patients came from Brighton, Eastbourne, and other places on the recommendation of their medical men. A large proportion of the patients who could not afford to pay came from the East-end of London. Evidence was then given as to the nursing staff.

Mr. Richard Smith, M.R.C.P., one of the medical staff, spoke in favour of a greater co-operation between general and special hospitals. He saw no reason why the staff on general should not belong to the staff of special hospitals. Special hospitals for women were much required, because of the want of accommodation for treating them at general hospitals. Serious operations in special diseases were certainly much better carried out in special than in general hospitals. For cancer and consumption special hospitals were necessary, but they were less requisite for children than for any other purpose. All the general hospitals now had special departments, but he considered that special hospitals for women were most essential. He certainly did not consider that the object of special hospitals had come to an end. It was difficult to get clinical instruction as to the diseases of women; but if it could be done, it would facilitate such instruction if special hospitals were done away with, and women were treated at general hospitals. That, however, would entail enormous extension.

Captain Hinckes, the next witness called, said he was Secretary to the Gordon Hospital in the Vauxhall Bridge Road, where patients were admitted free as well as by payment. It was started in 1884 to meet the requirements of persons with limited means who did not wish to go to general hospitals. The out-patient department was entirely free without governors' letters. The expenditure per bed was £105 in 1890, and for each patient £6 5s.

Dr. Whitmore, one of the founders of the hospital, stated that it was established more from philanthropic motives than from anything else, and he read one letter, which he stated was a sample of many he had received from patients. In answer to Lord Cathcart, the witness said that the name of the hospital had been altered, and its present title adopted with the sanction of the family of the late General Gordon, with whom he was on friendly terms.

On Thursday, April 30th, Mr. Frederick Wallace, F.R.C.S., a general practitioner, of Hackney and Shoreditch, gave evidence to the effect that nearly all the medical men of the neighbourhood were opposed to the provident plan which was adopted by the Metropolitan Hospital in Kingsland Road. The general practitioners had greatly suffered by the loss of some of their patients. Replying to Lord Thring, he said he could not reduce his fees to those charged at the dispensaries, as it would be *infra dig.*

Mr. G. O. M. Ryan, the Secretary of Queen Charlotte's Lying-In Hospital, and Dr. William Hope, the senior physician to the same establishment, gave evidence with reference to the operations of that institution. The latter gentleman stated that the mortality was greater amongst the unmarried patients than amongst the married ones, for many of the girls who came there were very young, ranging from 14 years of age to 25.

Lord Balfour of Burleigh, as President of the London Fever Hospital, gave a history of that institution up to the year 1871, prior to the establishment of the Metropolitan Asylums Board, during which time it really did the whole work of the metropolis. There were three classes of patients, viz., the ward patients, who paid £3 3s. for each case, private patients who paid £3 3s. per week, and patients sent from large firms, clubs, and hotels. These last contributed a certain amount, and their employés were taken in when attacked by fever, no further charge being made. He considered that the Fever Hospital was doing a useful work between two extremes. Patients who had large private houses could be isolated and treated in their own houses, and those at the opposite end of the scale went to the Metropolitan Asylums Board, while the Fever Hospital came in between and treated those who could pay partly, and private patients who paid a little more than their cost. It was an advantage to obtain the isolation which the Fever Hospital gave, not only to the sufferers themselves, but to the family and to the community. In times of pressure, or if there were an epidemic of any kind of fever in the metropolis, he was afraid the hospital would not be able to meet the demands upon it, and they were considering the propriety of extending it. They took in any kind of fever cases, but did not receive small-pox patients.

Major Christie, the Secretary, and Dr. Hopwood, the resident medical officer to the Fever Hospital, also gave evidence, and the Committee adjourned.

